# Differential Expression of Wnts after Spinal Cord Contusion Injury in Adult Rats

**DOI:** 10.1371/journal.pone.0027000

**Published:** 2011-11-02

**Authors:** Carmen María Fernández-Martos, Carlos González-Fernández, Pau González, Alfredo Maqueda, Ernest Arenas, Francisco Javier Rodríguez

**Affiliations:** 1 Laboratorio de Neurología Molecular, Hospital Nacional de Parapléjicos (HNP), Toledo, Spain; 2 Molecular Neurobiology Unit, MBB, Karolinska Institute, Stockholm, Sweden; University of Milan-Bicocca, Italy

## Abstract

**Background:**

Spinal cord injury is a major cause of disability that has no clinically accepted treatment. Functional decline following spinal cord injury is caused by mechanical damage, secondary cell death, reactive gliosis and a poor regenerative capacity of damaged axons. Wnt proteins are a family of secreted glycoproteins that play key roles in different developmental processes although little is known of the expression patterns and functions of Wnts in the adult central nervous system in normal or diseased states.

**Findings:**

Using qRT-PCR analysis, we demonstrate that mRNA encoding most Wnt ligands and soluble inhibitors are constitutively expressed in the healthy adult spinal cord. Strikingly, contusion spinal cord injury induced a time-dependent increase in Wnt mRNA expression from 6 hours until 28 days post-injury, and a narrow peak in the expression of soluble Wnt inhibitors between 1 and 3 days post-injury. These results are consistent with the increase in the migration shift, from day 1 to 7, of the intracellular Wnt signalling component, Dishevelled-3. Moreover, after an initial decrease by 1 day, we also found an increase in phosphorylation of the Wnt co-receptor, low-density lipoprotein receptor-related protein 6, and an increase in active β-catenin protein, both of which suffer a dramatic change, from a homogeneous expression pattern in the grey matter to a disorganized injury-induced pattern.

**Conclusions:**

Our results suggest a role for Wnts in spinal cord homeostasis and injury. We demonstrate that after injury Wnt signalling is activated via the Wnt/β-catenin and possibly other pathways. These findings provide an important foundation to further address the function of individual Wnt proteins in vivo and the pathophysiology of spinal cord injury.

## Introduction

Spinal cord injury (SCI) is a major cause of disability with no clinically accepted treatment [1]. The functional impairment following SCI is produced by multi-factorial processes as a result of primary mechanical damage, secondary cell death, reactive gliosis and the poor capacity to regenerate damaged axons. Typically, the epicentre of the SCI is characterized by necrotic neural death, while secondary tissue damage is also evident in the penumbra zone, where processes such as ischemia, hypoxia, excitotoxicity, free radical formation, protease release and inflammation contribute to the expansion of segmental loss of function. Another serious detrimental effect of SCI is the massive death of oligodendrocytes at a distance from the epicentre of the insult, leading to demyelination and deteriorated axon conduction. The final outcome is a pathohistological lesion that is far larger than the initial mechanical wound, consisting of a cyst cavity surrounded by a glial scar that inhibits axon growth [2]–[4].

The Wnt family of proteins plays key roles during the development of the nervous system, influencing cell proliferation and patterning, cell polarity and motility, axonal guidance, neuronal survival and connectivity, and cell-cell adhesion [5], [6]. This wide range of effects is possible because the specific responses elicited in target cells are dependent on the spatiotemporal distribution of Wnt ligands, modulators and receptors [7]–[11].

To date, 19 Wnt ligands and 10 Frizzled (Fz) receptors have been identified, which activate at least three different signalling pathways: the canonical or Wnt/β-catenin pathway; and the non-canonical Planar Cell Polarity (PCP, Wnt-JNK) and Wnt-Ca^2+^ pathways. Activation of the canonical pathway relies on the interaction of the Fz receptor with the low-density lipoprotein receptor-related protein 5/6 (LRP5/6), which leads to β-catenin stabilization in the cytosol, and ultimately in the nucleus, via Dishevelled (Dvl). Nuclear β-catenin combines with T-cell factor/lymphoid enhancer factor (TCF/LEF) family of DNA-binding proteins to activate the expression of genes that are mainly linked to cell proliferation [12]-[14]. By contrast, non-canonical pathways are activated by either LRP-independent Fz receptors or by a set of non-conventional receptors, such as Ryk and Receptor Tyrosine Kinase-Like Orphan Receptor (ROR-1/2), which have mainly been associated with cytoskeletal regulation and cell motility [15]-[17]. Finally, activation or inhibition of Wnt signalling is further modulated by co-receptors, such as Kremen (Krm1/2), and antagonists, such as the Wnt inhibitory factor 1 (Wif1), Dickkopf (Dkk) and secreted Frizzled-Related Proteins (sFRPs) [18], [19].

Although even the earliest reports suggested that Wnt expression in the nervous system may be prolonged into adulthood [20], [21], little is known about the expression or function of Wnt at these stages. Functional studies in the adult have been hampered by the labile nature of Wnt proteins, the embryonic lethality of mutants and by a lack of selective pharmacological tools [9]. Otherwise, the literature provides ample evidence implicating Wnt signalling pathways in adult CNS homeostasis and disease [13], [22]–[35], including SCI [32], [33], [36]–[41]. In this way, experimental modulation of Wnt-dependent pathways has produced promising results in different neuropathological situations [22], [23], [25], [29], such as in stroke, where Dkk1 is expressed by neurons with pro-apoptotic effects, a process that can be rescued by lithium [42]. Furthermore, Glycogen Synthase Kinase-3β (GSK3-β) inhibition [36], lithium [36], [43] or Wnt3a [41], all of which activate Wnt/β-catenin signalling, exert neuroprotective effects in SCI.

However, to our knowledge, the expression of Wnts has been reported as undetectable by *in situ* hybridization (ISH) in the adult spinal cord of mice, while after SCI only *Wnt1, Wnt4* and *Wnt5a* together with *Frizzled-1* and *Ryk* receptors were transiently induced [32], with no effect on ß-catenin mediated transcription [35]. On the other hand, in rats, the only Wnt protein shown to be expressed after SCI is Wnt5a, which is expressed by astrocytes from the glial scar and inhibits corticospinal regeneration through non-canonical Ryk activation [33].

Therefore, to investigate the role of the Wnt family of proteins in the adult spinal cord, we decided to profile by quantitative Real Time-PCR (qRT-PCR) the temporal pattern of mRNA expression for all Wnt ligands and inhibitors, followed by Western-blot and immunohistochemistry analysis of key members of the canonical pathway, in both non-injured and a moderate contusion model of SCI in rats. In agreement with previous indirect findings [22], [29], [44], [45], our results demonstrate that most Wnt ligands and inhibitors are constitutively expressed in the non-injured adult spinal cord, with activation of at least the canonical pathway in the grey matter. After injury, this pattern of expression was dramatically altered with significant activation of the canonical pathway in a large proportion of cells around the epicentre of the wound. The reasons for the difference between our results and previous studies in mice might be the higher sensitivity of qRT-PCR versus the ISH technique, but also a different pattern of Wnt expression between mice and rats. Hence, Wnts would appear to participate in the SCI pathophysiology.

## Materials and Methods

### Animals

Adult female Wistar rats, aged 10–12 weeks and weighing 200–220 grams were used throughout the study. Animals were obtained from Harlan (Barcelona, Spain) and housed in climate controlled quarters with a 12 hour light cycle. Handling was carried out according to European Union and NIH guidelines for animal experimentation in order to minimize suffering and the number of animals used. All the experimental protocols were approved by the Bioethics Committee of The National Hospital of Paraplegics (Permit numbers 51/2009 and 45/2008).

### Surgical procedure and experimental design

Sterile surgical techniques and methods were used throughout this study in a designated room. Briefly, rats were anesthetized with intraperitoneal injections of pentobarbital (40 mg/kg) and xylazine (10 mg/kg). Laminectomy was performed at the level of T8 via a controlled 200-kilodyne contusion injury using an Infinite Horizon Impactor (Precision Systems and Instrumentation LLC), and the overlying muscle and skin layers were sutured. After surgery, rats were allowed to recover on a warmed blanket with access to water and food, and they received daily subcutaneous injections of saline solution containing enrofloxacine (2.5 mg/kg) and buprenorphine (0.03 mg/kg) for the following 5 and 2 days, respectively. Post-operative care also included manual bladder emptying twice daily until recovery of voiding control was achieved, and inspection for signs of infection, dehydration or autophagia.

The Open-Field Locomotor Basso-Beattie-Bresnahan (BBB) scale was applied by two examiners blind to the treatments to determine severity and reproducibility of injury on days 1, 3, 7, 14 and 28 post-injury (dpi) [46]. In order to establish a homogeneous group in which expression levels were strictly due to temporal changes after injury, animals with a functional score 0–3 the day after surgery (at 24 hours) were excluded from the study. In the entire study, only one animal from the 28 dpi group was excluded. A total of 21 animals were randomly distributed between each of the following 7 groups for qRT-PCR analysis (n = 3 per group): Non-Injured Control (C), 6 hours post-injury (hpi), and 1, 3, 7, 14 and 28 dpi. For histological and Western blot studies 30 animals were randomly distributed into each of the following groups (n = 3 per group and technique): C, 1, 7, 14 and 28 dpi.

### RNA isolation and qRT-PCR analysis

At each of the time-points chosen for study, animals were terminally anesthetized with pentobarbital (40 mg/kg) and perfused intracardially with heparinized saline (150 ml) to remove blood from the tissue. Total RNA was isolated with the RNeasy Lipid Mini Kit (Qiagen) from a 1 cm long fragment of the spinal cord containing the wound epicentre, according to the manufacturer's instructions. Complementary DNA (cDNA) was synthesized from DNase-treated RNA (3 µg) as described previously [47]. For relative quantification, each gene of interest was first subjected to a serial dilution assay to determine the optimum detection range of Ct values, with a Ct threshold of 35 for undetectable levels of expression. Relative quantitation of all 19 Wnt ligands, the co-receptors of the β-catenin signalling cascade (LRP5/6), the soluble Wnt signalling inhibitors (sFRP-1/5, Wif1, Dkk-1/3) and the intracellular canonical Wnt signalling effectors (GSK-3β and β-catenin) was performed using 10 ng of reverse-transcribed total RNA, 20 pmol/ml of both sense and antisense primers, and the Fast SYBR Green PCR Master Mix (Applied Biosystems) in a final reaction volume of 20 µl. The reactions were run on an ABI PRISM 7900 Fast Sequence Detection System instrument and software (Applied Biosystem) according to the manufacturer's protocol.

To standardize the amount of sample cDNA added to the reaction, amplification of endogenous control 18S rRNA (primers sequence obtained from [48]) were included in a separate well as a real-time reporter. In all cases, primers were designed using Primer Express software and validated before their use on embryonic cDNA. Primers not specified in the attached table (Table 1) had previously been validated [47]. At the end of each run, melting curve profiles were performed to confirm amplification of specific transcripts. Relative quantification for each gene was performed by the ΔΔCt method [49] and reported as mean ± SEM of two separate determinations of 3 independent samples per experimental group.

**Table 1:** List of qRT-PCR primers.

Gene name	Forward primer	Reverse primer	Acc no.
β-catenin	5′-GCCAAGTGGGTGGCATAGA-3′	5′-TCCCTGTCACCAGCACGAA-3′	NM_053357.2
Dkk-1	5′-CTGTCTGCCTCCGATCATCA-3′	5′-CAGAAATGTCTTGCACAACACA-3′	NM_001106350.1
Dkk-2	5′-GCTCGCGGGCCAAAC-3′	5′-CAACTCCATCAAGTCC-3′	NM_001106472.1
Dkk-3	5′-CAGCTGTGACATCCAGACAGAAG-3′	5′-GCACCTGAAACTGTCATCTGAGA-3′	NM_138519.1
Dkk-4	5′-AGGCCTCTGTGGGCAACTT-3′	5′-CTAAGGCTGGGCTGCTGAGT-3′	NM_001109332.1
GSK-3β	5′-TCTGGCCACCATCCTTATCC-3′	5′-TTGCAGGCGGTGAAGCA-3′	NM_032080.1
sFRP-1	5′-CTGCCACCAGCTGGACAAC-3′	5′-ACCTTGCGCCCCATGA-3′	XM_224987.4
sFRP-2	5′-CGTGAAACGGTGGCAGAAG-3′	5′-CGGATGCTGCGGGAGAT-3′	NM_001100700.1
sFRP-3	5′-CTACCCTGGAACATGACCAAGAT-3′	5′-TGGCGTTAGCCTGGGTACTG-3′	NM_001100527.1
TCF-1	5′-GCTGCTTCAGGGCTAAGATTGT-3′	5′-TGTGACCTTGGCATGAGTTACG-3′	NM_012669.1
Wnt6	5̀-GCGGTCACTCAGGCCTGTT-3′	5′-GGGTGCCTGACAACCACACT-3′	NM_001108226.1
Wnt8	5′-CCTGGGAGCGGTGGAACT-3′	5′-CCTGGTGTGGGTTGAAAACTG-3′	NM_001106155.1
Wnt10b	5 -CCTCAAGCGCGGTTTCC-3′	5′-CAGCAGCCAGCATGGAGAA-3′	NM_001108111.1
Wnt15	5′-CACCCATGTGGGCATCAA-3′	5′-CCATGACACTTGCAGGTTGTTC-3′	NM_001107055.1
Wif1	5′-TGCGGTGCCCATGGA-3′	5′-CTGCCACGAACCCA-3′	NM_053738.1

Primers used for qRT-PCR analysis of the genes assessed here, including the gene symbol, primer sequence (forward and reverse sequence respectively) and GenBank accession number. The primers used to assess the expression of Wnt ligands and inhibitors not included in the list were obtained elsewhere [47].

### Western blotting

Spinal cords from animals intracardially perfused with heparinized saline were rapidly dissected and a 1 cm long fragment containing the injury epicentre was homogenized in RIPA buffer (Sigma-Aldrich) containing a proteinase inhibitor cocktail (Roche). Denatured protein samples (100 µg) from each group (C, 1, 7, 14 and 28 dpi: n = 3 per group) were resolved on a 10% SDS-PAGE gel (BioRad) and transferred to PVDF membranes (Millipore). Membranes were blocked for 1 hour in 5% non-fat dried milk containing TBS-T (0.1 M Tris-HCl [pH 7.4], 0.15 M NaCl and 0.1% Tween 20) and probed overnight at 4°C with a primary mouse Dvl-3 antibody (1∶500; Santa Cruz Biotechnology Inc.). After washing, the membranes were incubated for 1 hour at room temperature (RT) with a Horse Radish Peroxidase (HRP)-conjugated anti-mouse secondary antibody (1∶5000; Amersham), and the resulting antibody binding was visualized by bathing for 2 minutes in the ECL solution (SuperSignal West Pico Chemiluminescent Substrate, Thermo Scientific) and exposing to hyperfilm (Amersham) for 1–10 minutes. The antibodies were subsequently stripped from the membranes by incubating them for 5 minutes in buffer (0.1 M Glycine [pH 2.9]), and after washing in TBS-T for 15 minutes they were re-probed with a mouse anti-GAPDH antibody (1∶10.000; Abcam) as a loading control.

### Histology

As described above, 3 animals from each experimental group (C, 1, 7, 14 and 28 dpi) were perfused intracardially with a heparinized saline solution followed by 4% paraformaldehyde, and their spinal cords post-fixed for 4 hours in the same fixative. The spinal cords were then cryoprotected by immersion in 30% sucrose for 48 hours, embedded in Neg-50 frozen medium (Richard-Allan Scientific) and stored at −20°C. From each spinal cord, 30 µm thick parallel cryostat sections were obtained (Microm) from a 3-cm long fragment containing the wound epicentre, which were mounted on 33 serial slides and stored at −20°C.

To generate a histological time-course of phosphorylated-LRP6 (p-LRP6) and active β-catenin expression in the lesioned spinal cord, a set of parallel sections from each animal was processed by immunohistochemistry. Briefly, endogenous peroxidase activity was inactivated in the sections by incubation with 2% H_2_O_2_ in a 70% methanol solution at RT for 10 minutes, and they were then blocked for 1 hour at RT in blocking buffer (BB) containing 10% Fetal Bovine Serum (FBS), 0.3% Bovine Serum Albumin (BSA) and 0.3% Triton X-100 in TBS. The sections were then incubated overnight at 4°C with the primary rabbit anti-p-LRP6 (1∶100; Cell Signaling) or rabbit anti-active β-catenin (1∶500; Millipore) antibodies in BB, plus 1 hour at RT. Antibody binding was visualized by sequential incubations with a biotinylated anti-rabbit secondary antibody (1∶500; VectorLabs) and HRP-linked streptavidin (1∶500; Perkin Elmer), and with the “Nova Red Kit” (VectorLabs) according to the manufactureŕs instructions. The sections were finally dehydrated in graded ethanol, cleared with xylene and coverslipped with DPX (Panreac).

### Statistical analysis

Statistical comparisons were examined by *one-way ANOVA* (GraphPad Prism 4.0 software), followed by a *Tukey* post hoc test to determine individual differences between means. *P* values lower than 0.05 were considered as statistically significant.

## Results

### Wnt ligands expression is prolonged in the adult spinal cord and is modulated by injury

**Figure 1:**
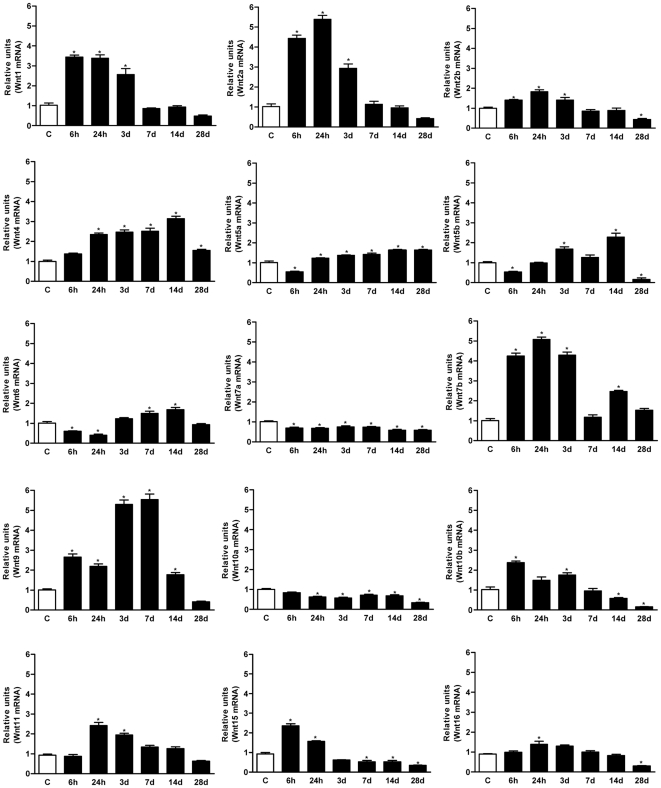
Wnt ligands expression in the spinal cord of adult rats following SCI. All Wnt ligands except for *Wnt3*, *3a*, *8a* and *8b* were constitutively expressed in the non-injured adult spinal cord. After SCI, 3 main patterns of Wnt ligand expression were observed: i) *No change or slight reduction* (*Wnt7a* and *10a*); ii) *early induction* (*Wnt1, 2a, 2b, 7b, 10b, 15* and *16*); and iii) *late induction* (*Wnt4, 5a, 5b, 6, 9* and *11*). All analyses were performed using total RNA samples isolated from a 1 cm long fragment of the spinal cord from non-lesioned control animals (C) and fragments containing the wound from contused animals at different times post-injury (6 and 24 hpi and 3, 7, 14 and 28 dpi). Expression of rat Wnt ligand genes was assessed by qRT-PCR using specific primers (Table 1 and [47]) and normalized to ribosomal *18S* expression. Values for each experimental group and day are expressed as mean ± SEM, n = 3. Each animal/sample (“n”) was measured in triplicate in two occasions (2 independent technical triplicates, 6 measurements per sample). *p<0.05 compared with C.

In order to investigate the role of the Wnt family of proteins in the adult spinal cord, we examined by qRT-PCR the temporal pattern of mRNA expression for all Wnt ligands in both non-injured rats and in SCI rats up to 28 days after moderate spinal cord contusion. Of the 19 Wnt ligands already described in rats, we found that most were expressed in non-injured adult spinal cord, only *Wnt-3, 3a, 8a* and *8b* mRNAs remained below the level of detection of the qRT-PCR protocol (Ct over 35). After injury, the Wnt ligand mRNA expression was regulated distinctly at the different time-points analyzed (Figure 1). The relative increases and decreases observed with respect to the non-injured basal value, allowed the ligands to be grouped in function of three distinct patterns: i) no change or a slight reduction (*Wnt7a* and *10a*); ii) early induction from 6 hours up to 3 dpi (*Wnt1, 2a, 2b, 7b, 10b, 15, 16*); and iii) late induction from 1–3 up to 7–14 dpi (*Wnt4, 5a, 5b, 6, 9, 11*). For instance, *Wnt1* and *2* are good examples of early induced genes that are markedly up-regulated up to 3 dpi, after which their mRNA expression decreases to control levels by 7 dpi. Conversely, *Wnt 5a, 5b* and *6* exhibited a 50% reduction in mRNA expression in the first 6 hpi, followed by a steady increase from 1–3 dpi to 14 dpi, when maximal expression was observed (Figure 1). Finally, none of the Wnt ligands with undetectable or null levels in non-injured spinal cords were expressed following injury.

### Wnt inhibitors and modulators are differentially expressed in the healthy and injured spinal cord

**Figure 2:**
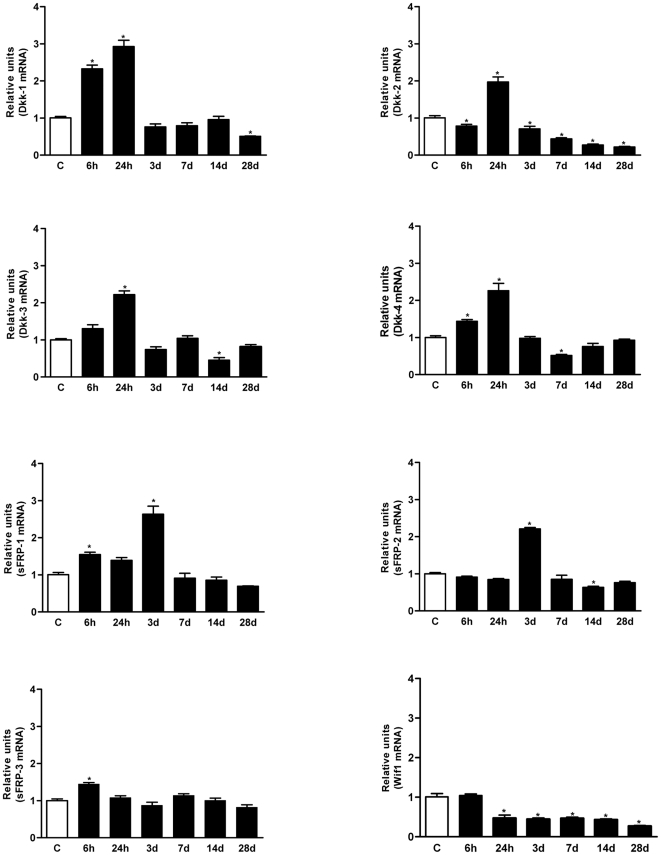
Short term up-regulation of Wnt inhibitors expression after SCI. Expression of the Wnt inhibitor genes *Dkk-1/4*, *sFRP* (*sFRP-1/5*) and *Wif1* was quantified by qRT-PCR as described in Figure1. Expression of all the inhibitors except *sFRP4* and *5* was detected in the non-injured spinal cord. After SCI, the *Dkks* were strikingly up-regulated with a narrow and specific peak after 24 hours, followed by a slightly delayed peak at 3 dpi for *sFRP-1* and *2*. By contrast, *Wif1* was dramatically down-regulated 24 hpi until the end of the study. Values for each experimental group and day are expressed as mean ± SEM, n = 3. Each animal/sample (“n”) was measured in triplicate in two occasions (2 independent technical triplicates, 6 measurements per sample). *p<0.05 compared with non-injured control animals (C*)*.

We next assessed the expression of secreted Wnt inhibitors and modulators, key regulators of extracellular signalling by the large and complex family of Wnt ligands. We focused our analysis on the families with well described roles in the nervous system: Dkk-1/4, sFRP-1/5 and Wif1. As observed for Wnt ligands, most of the Wnt inhibitors and modulators were expressed in the non-injured adult spinal cord, except *sFRP-4* and *sFRP-5* (Figure 2). Interestingly, the specific inhibitors of the canonical pathway, *Dkk-1/4*, were up-regulated early, with a striking peak of expression at 24 hpi that returned to basal levels at 3 dpi. The sFRPs are generally considered as broad and non-specific Wnt inhibitors, yet *sFRP-1* and *2* exhibited a similar but slightly delayed pattern of expression, with a narrow peak at 3 dpi, while *sFRP-3* remained stable. Finally, *Wif1*, a broad inhibitor of Wnt signalling, was dramatically down-regulated from 24 hpi until the end of the study.

### Canonical Wnt signalling is active in the adult spinal cord and in cells around the wound epicentre after SCI

**Figure 3:**
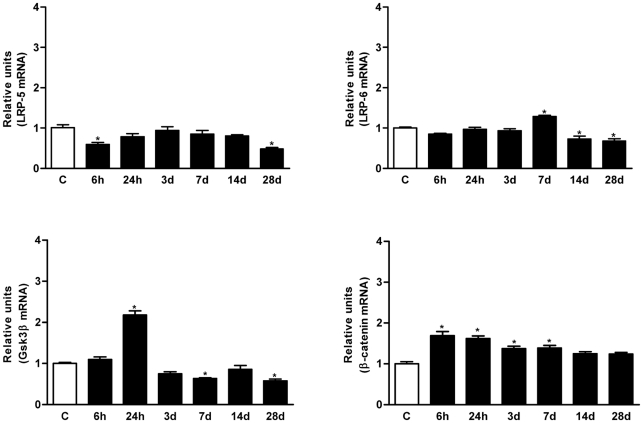
SCI alters the mRNA expression of canonical Wnt signalling components. Expression of the canonical co-receptor *LRP5/6*, the downstream intracellular inhibitory transducer *GSK3-ß* and the *ß-catenin* transcription factor were quantified by qRT-PCR as described in Figure1. All signalling components were expressed in non-injured spinal cord. SCI did not alter the transcription of the *LRP5/6* co-receptors, although there was a striking increase in *GSK3-β* expression that coincided with that of the *Dkk* inhibitors, as well as a mild up-regulation of *β-catenin* after 6 hours that gradually decreased until the end of the study. Values for each experimental group and day are expressed as mean ± SEM, n = 3. Each animal/sample (“n”) was measured in triplicate in two occasions (2 independent technical triplicates, 6 measurements per sample). *p<0.05 compared with non-injured control animals (C).

**Figure 4:**
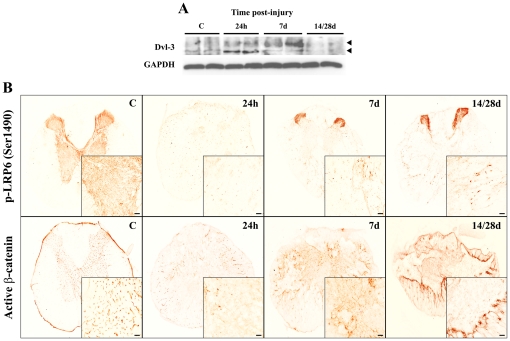
Active canonical Wnt signalling pathway in adult spinal cord before and after SCI. (**A**) Western blot of Dvl-3 with protein samples isolated from a 1 cm long spinal cord fragment from non-lesioned control animals (**C**) and SCI animals at 1, 7 and 14/28 dpi. SCI induced hyperphosphorylation (upper arrowhead) and thus, the activation of Dvl-3 24 hours post-injury, which peaked at 7 dpi. GAPDH levels were used as a control for protein loading. (**B**) Representative histological images of the expression of the active LRP6 canonical co-receptor (phosphorylated at serine 1490) and β-catenin (dephosphorylated at serine 37 and threonine 41) before SCI and at 1, 7 and 14/28 dpi. Active LRP6 and β-catenin were both expressed in the grey matter of the non-injured spinal cord, the latter exhibiting a vascular-like pattern. After SCI, active LRP6 and β-catenin was expressed in cells located in the white matter of the wound epicentre, with a clear increase from 7 dpi and a final location suggestive of a role in tissue response. Scale bars = 50 µm.

At the cellular level, Wnt binding to Fz or non-conventional receptors activates three distinct downstream signalling cascades: the canonical or Wnt/β-catenin; and the non-canonical Planar Cell Polarity (PCP, Wnt-JNK) and Wnt-Ca^2+^ pathways. As the non-canonical pathways remain poorly characterized, we focused on a representative set of components in the main canonical pathway. Irrespective of the Fz receptor expressed by a specific cell, activation of the canonical pathway requires the expression and recruitment (via serine phosphorylation) of LRP5/6 co-receptors [10], [50], [51]. Importantly, both receptors were expressed in non-injured adult spinal cord, a pattern which changed little for mRNA after SCI (Figure 3). Moreover, active LRP6 was homogenously distributed in the grey matter, although was also observed in several cells around the wound epicentre at 24 hpi, and it was even more prominent in the cells surrounding the cyst and in the grey matter from 7 dpi (Figure 4). Dvl is thought to be a key transducer of Wnt signalling [17] and it was expressed in a similar pattern to LRP6, with an increase in the active phosphorylated isoform 3 at 24 hpi peaking at 7 dpi (Figure 4).

Canonical activation relies on the dephosphorylation of β-catenin by GSK-3ß and the eventual translocation of the active β-catenin to the nucleus where it promotes transcription of a set of target genes after interacting with members of the TCF/LEF family [7], [9]. *β-catenin* mRNA expression was up-regulated following SCI, peaking at 6-24 hpi (a 1.8-fold increase when compared to the controls) and returning to basal levels at 14 dpi. In parallel, its negative regulator *GSK-3β* was also up-regulated at 6–24 hpi, its expression decreasing thereafter until the end of the study (Figure 3). Strikingly, active β-catenin was expressed strongly in the grey matter of the non-injured spinal cord with a highly suggestive vascular pattern. SCI altered this pattern, with cells in the white matter around the epicentre of the wound expressing β-catenin, increasing in number and occupying the centre of the wound at 7 dpi, and finally concentrating around the developing cyst at 14–28 dpi (Figure 4).

## Discussion

**Figure 5:**
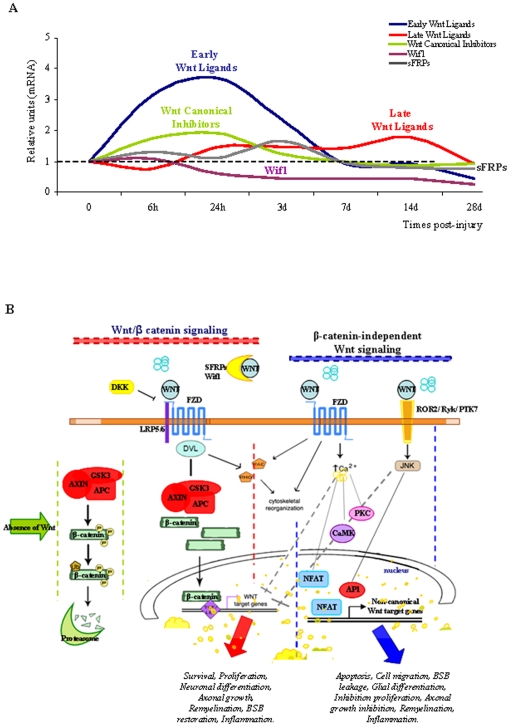
Temporal expression of Wnt mRNAs, and a schematic representation of Wnt signalling elements. (**A**) Summary of the integrated mRNA expression for early (*Wnt1, Wnt2a, Wnt2b*) and late Wnt Ligands (*Wnt4, Wnt5a/5b, Wnt6, Wnt7a, Wnt11*), as well as the Wnt Canonical Inhibitors (*Dkk-1/4*), sFRP (*sFRP-1/3*) and *Wif1*. (**B**) Representation of the Wnt/β-catenin and β-catenin-independent signalling pathways and their putative roles in SCI.

In the present study, we provide evidence that the expression of Wnt ligands, their inhibitors and components of their intracellular signalling pathways is prolonged in the adult spinal cord, as is the activation of the canonical pathway, suggesting that the Wnt family of proteins play a role in spinal cord function and physiology. More importantly, in a clinically relevant rat model of SCI we demonstrate that trauma induces a dramatic and time-dependent change in the physiological pattern of Wnt mRNA expression (Figure 5A). Furthermore, we describe a concomitant activation of Dvl-3, the downstream intracellular signalling transducer of Wnt, and activation of the canonical pathway in cells around the wound core in a pattern suggesting that it influences glial scar formation. To our knowledge, this is the first report demonstrating constitutive expression of Wnts in the adult spinal cord and their differential regulation after injury. These observations are likely to be highly relevant to understanding the potential role of Wnts in the cell and molecular responses induced by SCI (Figure 5B).

The clinical outcome of SCI can be improved by limiting the extent of secondary tissue damage, which is largely dependent on the inflammatory response induced during both the acute and chronic phases [2], [3], [52]. Minutes after injury, a massive inflammatory response is induced that is characterized by a variety of complex and interrelated events and cellular responses, in particular those involving astroglial and microglial reactivity or leukocyte infiltration. It was recently suggested that canonical Wnt signalling is involved in microglial proinflammatory instruction [28], while inhibitory and inductive roles have also been proposed for canonical and non-canonical signalling in proinflammation, respectively [53]–[56]. Indeed, canonical activation by lithium, Wnt1 or Wnt3a inhibits several inflammatory events, including endothelial activation [57], transendothelial migration of monocytes [58], and proinflammatory cytokine production by activated macrophages [54], [59]. By contrast, TLR/NFκB signalling promotes the production of proinflammatory cytokines and Wnt5a in an *in vitro* model of macrophage activation [53], [55], [56], [60], [61]. In turn, these factors exert an autocrine effect through the Fz5-mediated activation of the Wnt-Ca^2+^ non-canonical pathway, further augmenting the expression of pro-inflammatory genes. Thus, Wnt5a has been proposed to be one of the key macrophage-derived effector molecules that triggers and sustains chronic inflammation through both autocrine and paracrine signalling. Furthermore, the extent of the inflammatory response and that of the secondary cell death appears to correlate with the disruption of the blood spinal cord barrier (BSCB) [52], [62], [63], which may require the activation of β-catenin in endothelial cells in order to recover it the molecular and structural properties of a functionally mature BSCB [64]–[67]. Interestingly, the physiological vascular-like expression of active β-catenin protein was lost in the grey matter of the wound core, concomitant with an early peak in the expression of mRNA encoding canonical inhibitors like *Dkk* and *GSK-3β*, and the later expression of *Wnt5a* mRNA, which was previously shown to be expressed in reactive astrocytes of the glial scar [33]. Wnt5a expression has also been reported in other cell types, including fibroblasts [68] and endothelial cells [69], suggesting a cross-talk between all the cells involved in restoring tissue homeostasis after SCI, which based on our results may involve a larger number of ligands, receptors, and modulators of the Wnt family of proteins.

A serious consequence of SCI is the large-scale death of neurons and oligodendrocytes due to excitotoxic and inflammatory apoptosis [2], [3]. In this regard, in different neuropathological situations inhibition and activation of the canonical Wnt pathway has been shown to induce neuronal death and survival, respectively, including circumstances of excitotoxicity [23], [25], [70]–[72], brain ischemia [25], [43], [73] and Parkinson's [74] and Alzheimeŕs diseases [23], [45], [71], [75]–[77]. Indeed, lithium is a non-specific GSK-3ß inhibitor that is employed in the pharmacotherapy of bipolar diseases and it is a potent inhibitor of apoptotic neuronal death *in vitro*, as well as that associated with various neurodegenerative conditions *in vivo* [73], [78]–[81], including CNS stroke and SCI [36], [43]. This effect may be partly caused by the preservation and/or reinduction of the barrier properties of brain microvessels in the injured area. Importantly, acute administration of Wnt3a after moderate SCI provokes a significant recovery of motor function in association with a moderate neuroprotective effect [41]. Thus, Wnts would appear to fulfil a role of in adult CNS physiology as well as representing potential therapeutic targets.

Another critical impairment to functional recovery following SCI is the generation of a glial scar around the epicentre of the wound, which strongly inhibits axonal regeneration [3], [4]. As during development [6], [82], [83], Wnt proteins are critical factors governing axonal growth after CNS injury. Both endogenous Wnt2b and exogenous Wnt3a directly promote β-catenin-dependent CNS regeneration in the retina of adult mammals [84], [85], while transplantation of Wnt3a-secreting fibroblasts one week after SCI improves locomotor recovery by promoting axonal regeneration in rats [86]. Conversely, recent studies reported that SCI-induced Wnt5a expression around the injury site inhibited corticospinal axonal growth via non-canonical activation of the Ryk receptor in both mice [32] and rats [33]. This effect was overcome by intrathecal administration of Ryk neutralizing antibodies, enhancing the functional recovery. Accordingly, GSK-3β inhibition by lithium increases the intrinsic growth capacity of damaged neurons after SCI, permitting significant sprouting of descending corticospinal and serotoninergic axons in the caudal spinal cord and promoting functional recovery [37], [40]. On the other hand, glial scar is triggered by BSCB disruption and microglia/macrophage activation [4]. Indeed, this process appears to correlate with the areas of maximum BSCB breakdown and greatest number of activated microglia/macrophages [87]. Therefore, we can not exclude that at least in part Wnt promotion of axonal growth could be mediated by indirect action on BSCB restoration and inflammatory response modulation.

Wnts are also crucial physiological regulators of stem cells [13], [88], [89], which is significant as the adult spinal cord has been described to contain slow-dividing neural precursors that proliferate and differentiate into NG2+ glial progenitors after SCI, and they migrate around the lesion core to mainly form reactive astrocytes [90], [91]. β-catenin signalling is active in progenitor populations from adult neurogenic regions like the hippocampus, subventricular zone (SVZ) and olfactory bulb, and it is known to participate in the injury response of various tissues, including the CNS [26], [35], [84], [92]. Indeed, β-catenin has been shown to be responsible for SVZ/striatal proliferation after brain ischemia [93] and to be transcriptionally active in NG2 precursors associated to glial scar formation after traumatic brain injury [35]. However, the same authors describe a total lack of β-catenin transcriptional activation after SCI in mice, what could be derived of differences between CNS regions in front of the same type of insult [35] and/or versus to our results, a clear activation of both the canonical LRP6 co-receptor and β-catenin in cells around the injury core in a specific pattern that was highly suggestive of a role in glial scar formation, the reflect of a distinct physiopathology in rats [94] or intrinsic limitations of TCF-dependent transcriptional reporters [95], [96]. Intriguingly, a proportion of NG2+ positive precursors at a distance from the wound epicentre can differentiate into Olig2+ oligodendroglial precursors (OPC). β-catenin induced transcription is required for OPC instruction but following the inhibition for differentiation into mature oligodendrocytes during development and importantly adult remyelination [26], [97], [98]. Actually, there is considerable interest in Wnts with regards the development of novel stem cell based therapies [99], as Wnt3a increased both exogenous [100] and endogenous [41] neuronal differentiation of adult neural precursors.

In summary, our results provide compelling evidence that Wnts are expressed and transcriptionally regulated by SCI in adulthood. These novel findings provide an important foundation to further address the function of individual Wnt proteins *in vivo*, by loss and gain of function experiments, on the different cell populations of the healthy and injured adult spinal cord. In support, evidence in the literature already indicates that Wnts are not just mere bystanders of SCI. For instance, Wnt5a has been shown to be expressed by reactive astrocytes of the glial scar and play an inhibitory role on corticospinal regeneration through non-canonical Ryk activation [32], [33], while lithium [36], [43] or Wnt3a [41] induced Wnt/β-catenin signalling exerts neuroprotective effects in SCI. These findings, together with our data and increasing evidence linking Wnt signalling with neurodegenerative diseases in the adult [29] and in the development of CNS [6], suggest that the Wnt family of proteins might play a role in the pathophysiology of the SCI.
